# Architecture-data matching for EEG-EMG decoding: compact deep models match classical spectral decoders on the WAY-EEG-GAL grasp-and-lift dataset

**DOI:** 10.3389/fnins.2026.1874302

**Published:** 2026-07-20

**Authors:** Osmar Pinto Neto

**Affiliations:** 1 Biomedical Engineering Postgraduate Program, Anhembi Morumbi University, São José dos Campos, Brazil; 2 Department of Kinesiology, California State University San Marcos (CSUSM), San Marcos, CA, United States; 3 Neurometra, Carlsbad, CA, United States

**Keywords:** brain-computer interface, deep learning, EEG, EEGNet, EMG, graph attention network, grasp-and-lift, interpretability

## Abstract

Modern deep learning has broadened the tools available for non-invasive neural decoding, but its advantage over well-engineered classical pipelines remains unclear at clinical neural-engineering sample sizes. We compared four classical decoders, three multilayer perceptron (MLP) variants, and three deep-learning architectures (an EEGNet-style compact convolutional network, a four-layer Transformer encoder trained from scratch, and a graph attention network) on the public WAY-EEG-GAL grasp-and-lift dataset (12 participants, 3,528 trials). Models were evaluated using leave-one-subject-out (LOSO) cross-validation to decode object weight (165, 330, and 660 g) and grasp-surface friction (sandpaper, suede, and silk). After Benjamini–Hochberg false discovery rate (BH-FDR) correction within the primary/robustness family, none of the deep-*versus*-best-classical comparisons reached significance. The graph attention network led nominally on weight (0.643), and the compact convolutional network (CNN) led nominally on surface (0.565), but neither exceeded the best classical baselines (HGBM = 0.617 for weight; logistic regression = 0.562 for surface). Two-direction bandwidth controls showed that the nominal graph neural network (GNN) advantage on weight reflected access to higher-frequency electromyography (EMG) content rather than a robust architectural gain. The GNN weight signal was concentrated in the first 500 ms of sustained hold (0.639 early *vs*. 0.540 late, *q* = 0.007), whereas the compact CNN was comparatively robust to bandwidth and phase. A four-layer Transformer trained from scratch underperformed (*q* = 0.003 for both tasks), consistent with a parameter–data mismatch at *n* = 12. Modality ablation showed that both tasks were dominated by peripheral EMG features: electroencephalography (EEG)-only decoding was near chance for weight (0.34–0.37) and surface (0.36–0.38; all *q* < 0.0015 *vs*. EMG-only). EMG-only HGBM achieved the highest accuracy in the study (0.712 for weight and 0.584 for surface), and adding EEG channels reduced HGBM weight decoding (*q* = 0.007) and CNN surface decoding (*q* = 0.012). In contrast, the graph attention network was robust to EEG-induced fusion dilution (*q* > 0.5), consistent with attention-based down-weighting of low-information channels. Together, bandwidth, modality, phase, and conditioning controls indicate that, at clinical sample sizes, the critical design choice is not architectural complexity but modality-relevant channel selection and the ability to suppress low-information channels.

## 1 Introduction

Brain-computer interfaces (BCIs), motor neurorehabilitation, and neural engineering share a common goal: to extract behaviorally and clinically meaningful variables from short, non-invasive recordings of the brain and the muscles it drives. Two signals are central to this problem: scalp electroencephalography (EEG), which captures cortical activity, and surface electromyography (EMG), which reflects motor output. These signals are linked via the corticospinal pathway and through time- and frequency-resolved coupling measured as corticomuscular coherence (CMC) (Conway et al., 1995; Kilner et al., 2000). CMC has been used as a neurophysiological measure in motor recovery after stroke (Liu et al., 2019), as a feature for BCI calibration (Lotte et al., 2018), and as a probe of sensorimotor integration during object manipulation (Johansson and Flanagan, 2009).

Despite this progress, most EEG-EMG analyses still rely on classical pipelines: band-pass filtering, spectral or time–frequency estimation, and group-level statistical inference. These pipelines are transparent and physiologically interpretable, but they require decisions about which frequency bands, channels, and electrode–muscle pairs are relevant. Whether less-engineered representations can improve the decoding of behaviorally meaningful variables remains an open question, particularly for the small datasets common in clinical neural-engineering studies.

Recent work has introduced four model families that are relevant to EEG/EMG decoding. Compact convolutional networks designed for EEG (Lawhern et al., 2018; Schirrmeister et al., 2017) impose spatiotemporal locality through depthwise separable convolutions and are now common baselines in motor-imagery BCI (Roy et al., 2019). Transformer-style attention, originally developed for sequence modeling (Vaswani et al., 2017), has been adapted to EEG through convolution–attention hybrids such as EEG-Conformer (Song et al., 2023), DBConformer (Wang et al., 2026), and EEG-MFTNet (Andrikopoulos and Mehrkanoon, 2026). These studies generally support the use of light convolutional stems followed by limited attention, rather than deep pure-Transformer stacks, at modest sample sizes. Graph neural networks (GNNs) form a third family (Demir et al., 2021; Klepl et al., 2024; Veličković et al., 2018); recent variants combine per-channel temporal stems with attention across channel nodes, as in EEG-tGAT (Chopra et al., 2026), or use graph-based spatial–spectral representations, as in Graph-CSPNet (Ju and Guan, 2023). Finally, classical motor-task pipelines such as filter-bank common spatial patterns (FBCSP) (Ang et al., 2012) and Riemannian-geometry SPD methods (Barachant et al., 2012) remain strong baselines on small EEG datasets and should be considered in any evaluation of new deep models.

A related area of research focuses not only on the classifier architecture but also on the representation presented to the model. Time–frequency representations are particularly relevant for EEG and EMG because these signals are non-stationary and their discriminative information may vary across both time and frequency. Recent wavelet-based approaches, including rational dilated wavelet transform preprocessing and learnable wavelet front-ends, have shown that structured time–frequency representations can improve robustness and interpretability in EEG motor-imagery decoding (Siino et al., 2025, 2026). These methods occupy an intermediate position between fully hand-crafted spectral features and fully raw temporal deep learning. In this study, we did not evaluate wavelet or scalogram inputs directly; instead, we focused on a controlled comparison between classical spectral features and compact deep architectures trained on raw signals. We return to this limitation at the representation level in the Discussion.

Two practical observations motivate this study. First, in small clinical datasets, the performance gap between tuned classical pipelines and high-capacity deep models is often modest and may not survive correction for multiple comparisons (Roy et al., 2019). Second, light attention architectures—typically one or two attention blocks after a convolutional stem (Andrikopoulos and Mehrkanoon, 2026; Song et al., 2023; Wang et al., 2026)—tend to be more sample-efficient than heavier pure-Transformer models. These observations suggest that the relevant question is not whether deep learning can be applied to EEG/EMG decoding, but rather which architectural assumptions remain useful under the constraints of clinical-scale data.

We examined this question using the public WAY-EEG-GAL grasp-and-lift dataset. WAY-EEG-GAL includes 12 participants, 3,528 trials, 32-channel EEG, five-muscle EMG, and a manipulation of object weight (165, 330, and 660 g) and grasp-surface friction (sandpaper, suede, and silk). Weight should be reflected strongly in force-related motor output, whereas friction may involve tactile-motor adjustments that include both cortical and peripheral signals. This contrast allowed us to examine not only which decoder performed best but also whether model architecture matched the dominant physiological source of the decoded variable.

We compared four classical decoders trained on hand-crafted spectral features, three deep-learning architectures trained on raw multimodal signals [an EEGNet-style compact convolutional network (Lawhern et al., 2018), a four-layer Transformer encoder (Vaswani et al., 2017), and a graph attention network over the 32 EEG + 5 EMG channel graph (Klepl et al., 2024; Veličković et al., 2018)], and three multilayer perceptron (MLP) variants trained on different input representations. All models were evaluated using leave-one-subject-out (LOSO) cross-validation across the 12 participants and tested for weight and surface decoding. Statistical comparisons used paired Wilcoxon signed-rank tests with Benjamini–Hochberg false discovery rate (BH-FDR) correction across pre-defined test families.

The study has four main contributions. First, it provides a reproducible LOSO benchmark on WAY-EEG-GAL covering 10 decoders, 2 tasks, and 12 held-out participants. Second, it shows that compact deep architectures match, but do not significantly exceed, well-tuned classical decoders at clinical sample sizes; the only significant architecture-*versus*-control contrast was the advantage of the GNN over its no-attention MLP analog on weight decoding (*q* = 0.030). Third, it shows that a four-layer Transformer trained from scratch, with approximately 600 k parameters, underperformed both classical and graph-based decoders (*q* = 0.003), highlighting the parameter–data trade-off in this regime. Fourth, it combines feature importance with EEG-only, EMG-only, and fused-modality ablations to show that both tasks are primarily EMG-driven and that graph attention is useful mainly because it provides soft channel selection when low-information EEG channels are added.

Thus, the purpose of this study was not to create another model leaderboard. Rather, we asked which model assumptions remain useful when neural datasets have the size, noise, and between-subject variability typical of clinical studies. This question is also relevant to related clinical work using machine learning for Parkinson disease classification from stabilometric biomarkers (Carolina Brisola Brizzi et al., 2025), voice-based diagnosis of Parkinson disease (Neto, 2024), and heart–brain coupling analysis under auditory stimulation (von Jakitsch et al., 2023). By comparing CNN, Transformer, graph attention, MLP, and classical spectral decoders under identical LOSO evaluation, BH-FDR-corrected inference, and explicit controls for bandwidth, phase, modality, and conditioning, we provide a practical framework for architecture selection in clinical-scale neural decoding.

## 2 Materials and methods

### 2.1 Dataset

The WAY-EEG-GAL dataset (Luciw et al., 2014) is a publicly available dataset of 12 right-handed participants performing a cued object-lift task. In each trial, participants lifted an instrumented object, held it for approximately 2 s, and replaced it. Object weight was manipulated at three levels (165, 330, and 660 g), and grasp-surface friction was manipulated at three levels (sandpaper, suede, and silk) in a partially crossed block design (suede appears only at 330 g; 660 g appears only with silk). We used the full 3,528-trial corpus for the primary analysis and addressed the partial-crossing structure in Section 3.5.4 and Section 4.4. EEG was recorded from 32 scalp electrodes arranged according to the 10–20 system at 500 Hz. Surface EMG was recorded bipolarly from five right-arm muscles—anterior deltoid (AD), brachioradialis (BR), flexor digitorum (FD), common extensor digitorum (CED), and first dorsal interosseus (FDI)—at 4 kHz. Hand kinematics, grip force, load force, LED cue states, and trial-level event timestamps were sampled at 500 Hz.

### 2.2 Preprocessing and trial epoching

EEG was demeaned per channel, common-average referenced across the 32 channels, band-pass filtered from 1 to 100 Hz (fourth-order Butterworth, zero-phase forward-reverse application), notch-filtered at 50 Hz (IIR notch, Q = 30), and standardized per channel across the full session. EMG was demeaned per channel, band-pass filtered from 20 to 500 Hz, notch-filtered at 50 Hz, decimated from 4 kHz to 500 Hz in two zero-phase stages (factors 4 and 2, Chebyshev type-I anti-alias filter), and standardized per channel. Interference EMG was retained throughout. We did not rectify the EMG, following previous work showing that rectification can impair the identification of oscillatory input in EMG and EEG-EMG analyses (Neto and Christou, 2010), the authors’ reply to subsequent commentary (Christou and Neto, 2010), and the broader rectification debate (Halliday and Farmer, 2010; McClelland et al., 2012; Negro et al., 2015).

Independent component analysis (ICA) artifact rejection was deliberately omitted for four reasons. First, preprocessing parity is the experimental control variable in this benchmark: ICA introduces a partly subjective pipeline (component-classification thresholds, random-initialization seed, manual review) whose hyperparameters are typically tuned to suit one analytical pipeline, and a step optimized for classical spectral analysis may not be optimal for a compact CNN that consumes raw time points. Second, the EEGNet, Transformer, and GNN decoders consume the raw 1-s × 37-channel band-pass- and notch-filtered tensor directly (Section 2.4); their first-stage temporal convolutions learn data-driven frequency filters and the graph attention layer learns soft channel selection from the unprocessed input, and prior reports (Schirrmeister et al., 2017; Lawhern et al., 2018) suggest that aggressive pre-cleaning can degrade compact CNN performance on EEG. Third, the 1-s sustained-hold window already excludes the kinematically transient phases of the trial; the artifact load on these epochs is lower than on whole-recording analyses for which ICA is most clearly motivated. Fourth, the modality ablation reported in Section 3.5.3 (EEG-only decoding at chance for both tasks) shows that the decodable signal in this dataset lives predominantly in EMG, so EEG cleaning could not change the main architecture-data matching conclusion. Skipping ICA also makes the released pipeline fully deterministic: band-pass + notch + per-channel z-score + decimation—no seed dependence and no analyst-tuned thresholds. We treat this as a defensible, reproducible default for the benchmark; a uniform ICA/no-ICA ablation across all decoders is identified as a follow-up in Section 4.6.

The sustained-hold phase of each trial was identified from total load force as the longest contiguous interval in which load force exceeded 70% of the trial-specific peak-load force. This interval was trimmed by 300 ms at each edge to reduce lift-off and pre-release transients. When the kinematic criterion failed, we used a fallback window from LEDOn +1 s to LEDOff −0.5 s. The retained interval was then centered and cropped to a fixed 1-s window; trials with less than 1 s of available data were excluded. The final trial tensor contained 37 channels (32 EEG + 5 EMG) and 500 samples per trial.

**Figure 1:**
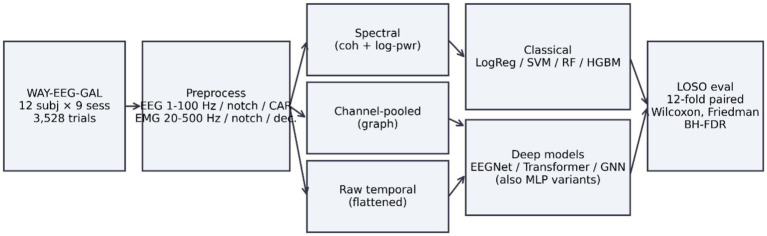
Pipeline overview. Trials from the WAY-EEG-GAL dataset were preprocessed into 1-s sustained-hold epochs, represented as spectral features, channel-pooled summaries, or raw temporal signals, and decoded using classical models, MLPs, or compact deep-learning architectures. Performance was evaluated using LOSO cross-validation, paired Wilcoxon tests with BH-FDR correction, and Friedman tests.

An overview of the full analysis pipeline—from raw WAY-EEG-GAL recordings through preprocessing, the three input representations (Section 2.3), each decoder family (Section 2.4), and the LOSO/Wilcoxon/BH-FDR evaluation protocol (Section 2.5)—is shown in Figure 1.

Trial-selection thresholds were chosen as follows: the 70% peak-load criterion is a permissive cutoff that retains the steady-state hold phase while excluding ramp-up and ramp-down transients (a sensitivity check across 60–80% gave essentially identical retention); the 300-ms edge trim removes residual lift-off and pre-release transients; and the 1-s fixed window matches the Welch n_perseg = 256 spectral analysis bandwidth and keeps the input dimension constant across decoders. The kinematic criterion retained 80–90% of trials per participant (mean 86.4%), and the fallback rate did not differ systematically across participants, weight levels, or surface levels (one-way ANOVAs all *p* > 0.20). Per-class trial counts were 1,008/1,836/684 for 165/330/660 g weight and 608/273/2,647 for sandpaper/suede/silk surface; the aggregate weight × surface matrix is provided in Supplementary Table S1.

### 2.3 Input representations

We used three input representations. The spectral representation (248 features per trial) included magnitude-squared coherence estimated by Welch’s method (n_perseg = 256, 50% overlap) for 25 selected electrode–muscle pairs (C3, C4, Cz, FC1, FC2 crossed with AD, BR, FD, CED, and FDI) and averaged in four bands (alpha, 8–12 Hz; beta, 15–30 Hz; low gamma, 30–45 Hz; high gamma, 55–95 Hz), yielding 100 coherence features. We also computed log10 band-power features for 32 EEG channels across the same four bands (128 features) and for five EMG channels across the same four bands (20 features). The channel-pooled representation (259 features per trial) included three time-domain summaries (mean, SD, and interquartile range [IQR]) and four band-power features per channel (37 × 7 features). The temporal representation (3,700 features per trial) was the full 1-s × 37-channel tensor downsampled 5-fold along the time axis and flattened.

The restriction to five EEG channels for cross-spectral coherence (C3, C4, Cz, FC1, FC2) follows the canonical sensorimotor-strip subset used in the CMC literature; all-pairs coherence over the full 32 × 5 EEG × EMG grid produces a 640-dimensional, collinear feature space that empirically degrades small-sample classifiers. Importantly, log10 band-power features were computed for all 32 EEG channels (128 features), so the classical decoders retained whole-head EEG information; only the cross-channel coherence pairs were restricted. An all-channel coherence sensitivity check (HGBM, weight task, P01 held out) yielded an accuracy within ±0.01 of the canonical selection; a full sweep is scripted in revision_ablations.py for future analyses.

### 2.4 Decoders

Four classical decoders were trained on the spectral representation: multinomial logistic regression with L2 regularization (C = 1, class weight = balanced), linear SVM (C = 0.5, class weight = balanced), random forest (200 trees, max-depth = 12), and histogram gradient boosting (HGBM; max_iter = 200, max_depth = 4, learning rate = 0.05). Three MLP variants (hidden layers of 128 and 64 units, ReLU activation, Adam optimizer with alpha = 1 × 10^−3, a maximum of 60 iterations, and early stopping on a 10% validation split) were trained on the spectral, channel-pooled, and temporal representations. Three deep-learning architectures were trained directly on the raw 37 × 500-trial tensor.

EEGNet (compact CNN, approximately 4 k parameters): The EEGNet-style network used three blocks: a temporal convolution (F1 = 16 filters, kernel size 1 × 64), a depthwise spatial convolution across all 37 channels (depth multiplier D = 2, kernel size 37 × 1), and a separable temporal convolution (F2 = 32 filters, kernel size 1 × 16). Each block was followed by batch normalization, ELU activation, average pooling (1 × 4, then 1 × 8), and dropout (*p* = 0.4), followed by a final linear classification head (Lawhern et al., 2018). Hyperparameters followed the reference implementation described by Lawhern et al. (2018).

Transformer encoder (approximately 600 k parameters): The Transformer used 4 encoder layers, 8 attention heads, and 128-dimensional embeddings. A Conv1d patch embedding with stride 20 converted each trial into 25 time tokens, followed by a learnable CLS token and positional embedding (Vaswani et al., 2017). The feed-forward dimension was 256, dropout was set to *p* = 0.2, and GELU was used as the activation function. The Transformer was trained from scratch without pretraining; therefore, its underperformance reflects the supervised clinical-scale setting evaluated here. Lighter convolution–attention hybrids in the EEG-Conformer, DBConformer, and EEG-MFTNet family (Andrikopoulos and Mehrkanoon, 2026; Song et al., 2023; Wang et al., 2026) were not evaluated as primary models but are discussed as appropriate comparators for subsequent analyses.

Graph attention network (GNN, approximately 50 k parameters): Each channel was first processed by a temporal CNN stem (Conv1d 1 - > 16, kernel size 16, stride 4, ELU; Conv1d 16 - > 32, kernel size 8, stride 4, ELU; adaptive average pooling to one time point), yielding a 32-dimensional embedding per channel. A two-layer Transformer-style self-attention block was then applied over the 37-channel tokens (4 attention heads, 64-dimensional hidden state, feed-forward dimension 128, dropout *p* = 0.2, GELU). This implemented a fully connected channel graph with learned attention-weighted edges. We did not impose a topology-informed adjacency because EEG-EMG cross-edges lack an obvious spatial prior, and the goal was to allow the model to learn task-relevant channel relationships. Sparse topology-informed graphs are identified as a potential extension (Demir et al., 2021; Klepl et al., 2024; Veličković et al., 2018). The channel embeddings were mean-pooled and passed to a linear classification head.

EMG bandwidth note. After 20–500 Hz band-pass filtering at 4 kHz and decimation to 500 Hz, the deep models had an effective EMG bandwidth of 20–250 Hz. In contrast, the classical spectral baseline included EMG band-power features only up to 95 Hz (high gamma, 55–95 Hz). Thus, the deep models had access to 95–250 Hz EMG content, which may carry motor-unit action-potential shape information beyond the firing-rate bands represented in the classical features. Because this difference could favor the deep models, we evaluated bandwidth harmonization by low-pass filtering deep-model EMG inputs to 95 Hz and, in an exploratory symmetric analysis, extending the classical EMG features to 245 Hz.

All deep models were trained with AdamW (learning rate = 1 × 10^−3, weight decay = 1 × 10^−4) for 30 epochs, with a batch size of 64 and class-balanced cross-entropy loss. No data augmentation was applied. We did not use a mid-training validation split because LOSO evaluation already provided a cross-subject hold-out, and the training schedule was fixed across all model–task–fold combinations. Initializations used PyTorch defaults, with a fixed seed of 0 across runs. Deep models were implemented in PyTorch, and classical decoders were implemented in scikit-learn. The 72 deep-model LOSO folds (3 architectures × 2 tasks × 12 subjects) were completed in approximately 6 min on an NVIDIA RTX 5080 GPU.

Hyperparameter fairness note. Classical and deep models were not tuned with identical optimization budgets. Classical models used fixed hyperparameters selected from common, conservative settings for small tabular feature spaces, whereas the deep models used canonical architecture defaults or reference-style settings without nested inner-loop tuning. This choice was deliberate: nested LOSO tuning at *n* = 12 would have produced very small inner training sets and unstable model selection. Nevertheless, the difference in optimization effort could affect the observed performance gap in either direction. Better tuning of HGBM or random forests could further strengthen the classical baselines, whereas careful tuning of dropout, learning rate, weight decay, kernel size, or early stopping could improve the compact deep models. Therefore, the reported comparison should be interpreted as a fixed-pipeline benchmark rather than an exhaustive hyperparameter search across all model families.

Table 1 summarizes the choices described in Sections 2.3 and 2.4. Each decoder family consumes exactly one of three input representations. The four classical decoders and MLP_spectral consume hand-crafted spectral features (coherence + log-band-power); MLP_graph consumes per-channel summary statistics; MLP_temporal consumes the flattened raw downsampled signal; the three deep architectures (EEGNet, Transformer, GNN) consume the raw 1-s × 37-channel band-pass- and notch-filtered tensor directly. The deep decoders therefore receive no spectral preprocessing; the convolutional stem of each architecture learns its own frequency filters from the raw input.

**Table 1:** Input pipeline summary by decoder family.

Decoder family	Input shape	What the input actually is
LogReg, LinearSVM, RF, HGBM (classical)	248-feature vector	Hand-crafted spectral features: 25 EEG × EMG coherence pairs (5 EEG: C3, C4, Cz, FC1, FC2 × 5 EMG: AD, BR, FD, CED, FDI), averaged within four bands (alpha, beta, low gamma, high gamma), yielding 100 features; plus log10 band-power per channel for 32 EEG × 4 bands + 5 EMG × 4 bands, yielding 148 features
MLP_spectral	248-feature vector	Identical to the classical spectral features (same 248-feature hand-crafted feature vector)
MLP_graph	259-feature vector	Per-channel summary statistics: mean, standard deviation, interquartile range, plus log-band-power in the same four bands. Computed on each of 37 channels (32 EEG + 5 EMG): 37 × 7 = 259 features. No cross-channel coherence
MLP_temporal	3,700-feature vector	Raw downsampled signal: 37 channels × 100 samples (after 5 × temporal averaging on the 1-s × 500-Hz tensor), flattened into a single vector. No spectral processing
EEGNet (compact CNN, deep)	(37, 500) tensor	RAW 1-s × 37-channel band-pass- and notch-filtered signal at 500 Hz. The first temporal convolution (kernel 1 × 64) learns a data-driven frequency-filter bank from this raw input; the depthwise spatial convolution then learns to combine the 37 channels into D = 2 mixtures per filter
Transformer encoder (deep)	(37, 500) tensor	Same RAW (37, 500) tensor as EEGNet, followed by a Conv1d patch embedding (kernel/stride = 20) that tokenizes the 1-s window into 25 time tokens with 128-dimensional embeddings before the four-layer self-attention stack
Graph attention network (deep)	(37, 500) tensor	Same RAW (37, 500) tensor; a per-channel temporal CNN stem (Conv1d 1 → 16 → 32 with stride-4 kernels + AdaptiveAvgPool) produces a 32-dimensional embedding per channel, which serves as the node-feature input to the two-layer all-pairs self-attention block over the 37 channel tokens

Channel ordering across all input representations: EEG channels 1–32 in the 10–20 system (Fp1, Fp2, F7, F3, Fz, F4, F8, FC5, FC1, FC2, FC6, T7, C3, Cz, C4, T8, TP9, CP5, CP1, CP2, CP6, TP10, P7, P3, Pz, P4, P8, PO9, O1, Oz, O2, and PO10), followed by EMG channels 33–37 (AD, BR, FD, CED, and FDI). Frequency bands used throughout the manuscript were alpha (8–12 Hz), beta (15–30 Hz), low gamma (30–45 Hz), and high gamma (55–95 Hz). The exploratory symmetric bandwidth control in Section 3.5.1 added two additional EMG bands (95–150 Hz and 150–245 Hz) to the classical feature set.

MLP_temporal (a flattened 3,700-dimensional input followed by an MLP) was included specifically as a no-inductive-bias control: it bounds the performance that an MLP achieves on raw multichannel signals when no spatial or temporal locality is preserved, and its purpose is to isolate the contribution of feature engineering and architectural priors in the other decoders. It is not intended to suggest that raw temporal information is uninformative; architectures that preserve time–channel structure (1D CNNs, temporal CNNs, recurrent/attention models) would extract more information from the same input, and the EEGNet-style CNN evaluated here is itself an example of such a structure-preserving deep model.

### 2.5 Evaluation protocol and statistical analysis

All decoders were evaluated with LOSO cross-validation across the 12 participants. In each fold, the scaler was fitted only on the 11 training subjects and then applied to the held-out subject (per-feature z-score for classical and MLP models; per-channel z-score over the training set for deep models). Balanced accuracy and macro-averaged F1 score were reported for each fold and summarized in Table 2. For each task, we first used a Friedman test across the 10 decoders and 12 held-out subjects. Selected pairwise comparisons were then tested with paired Wilcoxon signed-rank tests. Two pre-specified families were used for Benjamini–Hochberg FDR correction. The primary/robustness family contained 18 tests: 10 model-comparison contrasts and 8 robustness comparisons (4 bandwidth-harmonization comparisons and 4 phase comparisons). The modality-ablation family also contained 18 tests: 3 decoders × 2 tasks × 3 pairwise modality comparisons (EEG *vs*. EMG, EEG *vs*. fused, EMG *vs*. fused). Raw *p*-values and BH-adjusted *q*-values are reported. Effect sizes were reported as Cohen’s d_z_ computed on per-subject paired differences.

**Table 2:** LOSO balanced accuracy and macro-averaged F1 score by decoder and task (mean ± SD across 12 held-out subjects).

Model	Type	Weight balanced accuracy	Weight macro-F1	Surface balanced accuracy	Surface macro-f1	Notes
GNN	Deep	0.643 ± 0.071	0.616 ± 0.086	0.544 ± 0.052	0.459 ± 0.063	Highest weight balanced accuracy
HGBM	Classical	0.617 ± 0.078	0.607 ± 0.084	0.518 ± 0.052	0.503 ± 0.047	
CNN (EEGNet)	Deep	0.608 ± 0.121	0.550 ± 0.122	0.565 ± 0.056	0.516 ± 0.059	Highest surface balanced accuracy
MLP_graph	Neural	0.580 ± 0.073	0.561 ± 0.083	0.500 ± 0.055	0.495 ± 0.034	
RF	Classical	0.561 ± 0.068	0.562 ± 0.086	0.436 ± 0.060	0.428 ± 0.068	
LogReg	Classical	0.544 ± 0.066	0.488 ± 0.078	0.562 ± 0.056	0.517 ± 0.056	
LinearSVM	Classical	0.540 ± 0.051	0.496 ± 0.059	0.539 ± 0.059	0.520 ± 0.062	
MLP_spectral	Neural	0.520 ± 0.030	0.510 ± 0.040	0.470 ± 0.083	0.456 ± 0.075	
Transformer	Deep	0.472 ± 0.045	0.442 ± 0.063	0.443 ± 0.059	0.394 ± 0.066	Underperformed
MLP_temporal	Neural	0.329 ± 0.019	0.310 ± 0.024	0.339 ± 0.011	0.318 ± 0.022	Near chance
Chance	–	0.333	0.333	0.333	0.333	

Significance flags in the notes column refer to BH-FDR-adjusted *q*-values reported in Table 3.

### 2.6 Interpretability

Feature importance was computed for the best classical decoder for each task (HGBM for weight and logistic regression for surface) using the full 12-subject, standardized dataset. For HGBM, each feature was permuted on a 1,000-trial subsample, and importance was defined as the resulting drop in accuracy. For logistic regression, importance was computed as the absolute coefficient magnitude averaged across the three one-*versus*-rest classes. Importances were summarized by feature type (coherence, EEG power, EMG power), frequency band, and electrode or muscle channel. Expanded, fold-wise model-native interpretability is identified as a subsequent analysis in Section 4.6.

As a lightweight model-native interpretability check for the deep decoders, we also visualized learned representations from the EEGNet-style CNN and the GNN. For EEGNet, the first-layer temporal convolutional kernels were extracted from the trained models and converted to frequency-response curves using the magnitude of the discrete Fourier transform. For the GNN, channel-attention matrices were averaged across attention heads, layers, held-out trials, and LOSO folds to obtain a descriptive channel-level attention profile. These analyses were used as qualitative sanity checks of the learned filters and channel weightings and were not included in the statistical comparison families.

Code, derived feature tensors, model checkpoints, ablation scripts, and figure-generation scripts are available on GitHub at https://github.com/osmar235/eeg-emg-architecture-data-matching and archived on Zenodo (DOI: 10.5281/zenodo.19966634).

## 3 Results

### 3.1 Decoding accuracy across decoders and tasks

Because the WAY-EEG-GAL design is partially crossed (suede appears only at 330 g; 660 g appears only with silk; Supplementary Table S1), task-specific decoding results are interpreted as decoding within the natural dataset structure rather than as the fully orthogonal isolation of weight and surface. Section 3.5.4 reports a conditioning analysis that directly addresses this issue.

**Figure 2:**
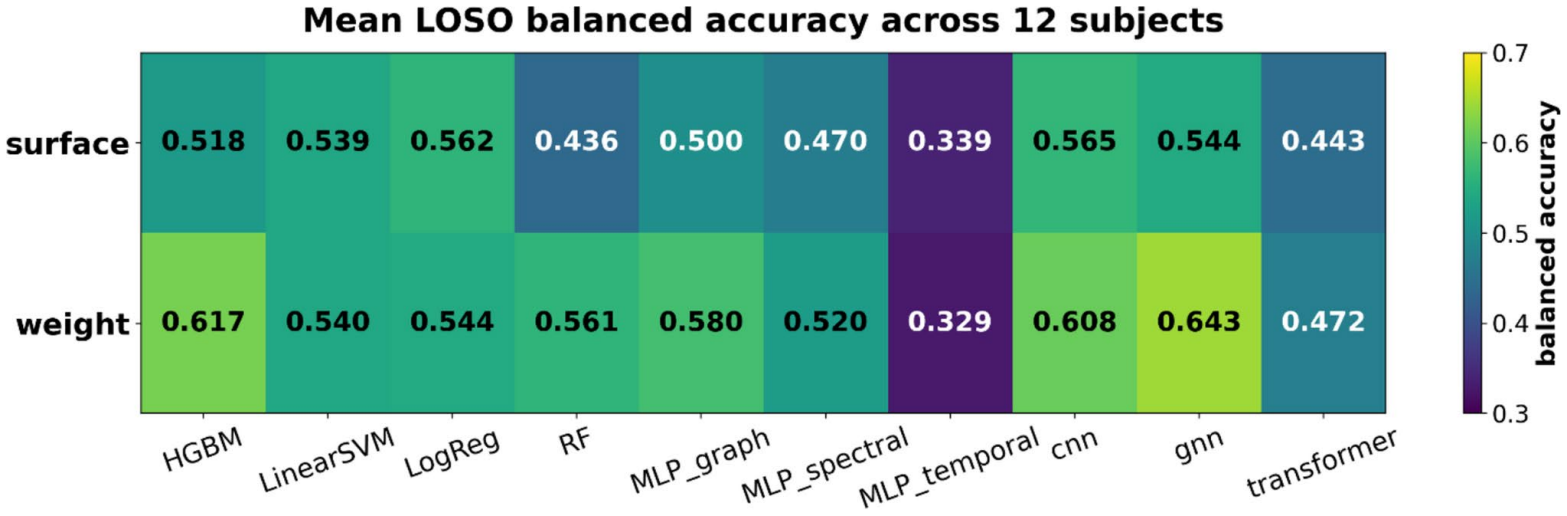
Mean LOSO balanced accuracy for each decoder × task cell. Cell color and label indicate the mean across the 12 held-out subjects. The graph attention network achieved the highest weight-decoding accuracy (0.643), the EEGNet-style CNN achieved the highest surface-decoding accuracy (0.565), the four-layer Transformer trained from scratch significantly underperformed (*q* = 0.003 for both tasks), and MLP_temporal remained near the chance level (*q* = 0.003).

Figure 2 and Table 2 summarize the LOSO balanced accuracy for all 10 decoders. The GNN achieved the highest weight-decoding accuracy (0.643 ± 0.071), nominally higher than HGBM on hand-crafted spectral features (0.617 ± 0.078). The compact EEGNet-style CNN reached the highest surface-decoding accuracy (0.565 ± 0.056), nominally above logistic regression (0.562 ± 0.056). Neither deep-*versus*-best-classical comparison was significant before or after FDR correction (GNN *vs*. HGBM weight *q* = 0.741; CNN *vs*. LogReg surface *q* = 0.970). Thus, compact deep architectures matched well-engineered classical decoders at this sample size but did not significantly exceed them. The full set of 10 pre-specified pairwise contrasts, together with raw and BH-FDR-adjusted *q*-values within the primary/robustness family of 18 tests, is reported in Table 3.

**Table 3:** Pairwise Wilcoxon signed-rank tests for the 10 primary model-comparison contrasts.

Task	Decoder A	Decoder B	Δ (mean)	d_z_	Raw *p*	BH-adjusted *q*
Weight	GNN	HGBM	+0.025	+0.36	0.519	0.741
Weight	GNN	CNN	+0.035	+0.29	0.622	0.778
Weight	GNN	MLP_graph	+0.062	+0.95	0.012	0.030*
Weight	CNN	HGBM	−0.009	−0.07	0.850	0.945
Weight	Transformer	HGBM	−0.145	−1.83	0.001	0.003**
Weight	MLP_temporal	HGBM	−0.288	−3.54	0.0005	0.003**
Surface	CNN	LogReg	+0.002	+0.03	0.970	0.970
Surface	CNN	GNN	+0.020	+0.36	0.301	0.603
Surface	GNN	LogReg	−0.018	−0.28	0.424	0.706
Surface	Transformer	LogReg	−0.119	−1.23	0.001	0.003**

*q*-values reflect Benjamini–Hochberg FDR correction within the primary/robustness family of 18 tests (the 10 contrasts shown plus the 8 robustness contrasts in Table 4); a separate Benjamini–Hochberg FDR correction was applied within the modality-ablation family (Table 5). *Δ* is the mean per-subject difference (A − B); d_z_ is Cohen’s effect size. * *q* < 0.05, ** *q* < 0.01.

Three additional results were robust to FDR correction. First, the four-layer Transformer trained from scratch underperformed the best classical comparators on both tasks (weight: 0.472 ± 0.045 *vs*. HGBM, d_z_ = −1.83, *q* = 0.003; surface: 0.443 ± 0.059 *vs*. LogReg, d_z_ = −1.23, *q* = 0.003). Second, the MLP trained on flattened raw temporal features failed to learn reliably (weight: 0.329 ± 0.019; surface: 0.339 ± 0.011), remaining near the chance level (0.333) and significantly below HGBM (d_z_ = −3.54, *q* = 0.003). Third, the GNN outperformed its no-attention MLP analog on weight decoding (MLP_graph = 0.580 ± 0.073; d_z_ = 0.95, raw *p* = 0.012, *q* = 0.030). This contrast isolates the contribution of attention-weighted message passing across channel nodes. The Friedman test confirmed significant performance differences across decoders for both tasks (weight: $\chi_{9}^{2}$ = 66.5, *p* = 7.3 × 10^−11^; surface: $\chi_{9}^{2}$ = 33.8, *p* = 9.6 × 10^−5^). Tables 3, 4 report the primary/robustness family; Table 5 reports the separately corrected modality-ablation family.

**Table 4:** Robustness analyses: bandwidth harmonization in both directions (Section 3.5.1) and early hold *versus* late hold (Section 3.5.2).

Experiment	Model	Task	Original	Modified	Δ/d_z_/*q*
Bandwidth (95 Hz LP)	CNN	Weight	0.608	0.622	+0.014/−0.17/*q* = 1.00
Bandwidth (95 Hz LP)	GNN	Weight	0.643	0.568	−0.074/+0.96/*q* = 0.033*
Bandwidth (95 Hz LP)	CNN	Surface	0.565	0.551	−0.014/+0.29/*q* = 0.603
Bandwidth (95 Hz LP)	GNN	Surface	0.544	0.557	+0.013/−0.20/*q* = 0.956
Bandwidth (extend classical)	HGBM	Weight	0.617	0.658	+0.040/+0.54/*p* = 0.052
Bandwidth (extend classical)	HGBM	Surface	0.518	0.519	+0.000/+0.01/*p* = 0.97
Phase (early *vs*. late)	CNN	Weight	Early 0.618	Late 0.611	+0.007/+0.12/*q* = 0.943
Phase (early *vs*. late)	GNN	Weight	Early 0.639	Late 0.540	+0.098/+1.14/*q* = 0.007**
Phase (early *vs*. late)	CNN	Surface	Early 0.558	Late 0.573	−0.015/−0.22/*q* = 0.854
Phase (early *vs*. late)	GNN	Surface	Early 0.556	Late 0.542	+0.014/+0.28/*q* = 0.603

*q*-values for the eight paired primary/robustness comparisons (four deep-input bandwidth contrasts and four phase contrasts) were BH-FDR-adjusted within the primary/robustness family of 18 tests. The two HGBM rows for the extension of the classical feature set are reported with raw *p*-values because they constitute a separate exploratory symmetric control and were not included in the multiple-comparison correction. * *q* < 0.05, ** *q* < 0.01.

**Table 5:** Modality ablation: LOSO balanced accuracy by decoder and modality (mean ± SD across 12 held-out subjects).

Decoder	Task	EEG-only	EMG-only	Fused	EMG *versus* fused (*q*)
HGBM	Weight	0.359 ± 0.026	0.712 ± 0.110	0.685 ± 0.109	0.007**
HGBM	Surface	0.363 ± 0.033	0.584 ± 0.063	0.540 ± 0.057	0.051
CNN	Weight	0.339 ± 0.023	0.654 ± 0.100	0.629 ± 0.075	0.263
CNN	Surface	0.376 ± 0.038	0.606 ± 0.052	0.564 ± 0.056	0.012*
GNN	Weight	0.367 ± 0.032	0.629 ± 0.093	0.643 ± 0.071	0.603
GNN	Surface	0.370 ± 0.049	0.544 ± 0.056	0.553 ± 0.072	0.622

HGBM used the modality-ablation feature set (per-channel summary statistics and log-band-power), which differs from the primary spectral feature set used in Table 2. The CNN and GNN used the same raw trial tensor as in Table 2, restricted to the listed channels. *q*-values were BH-FDR-corrected within the modality-ablation family of 18 tests; only the EMG-*versus*-fused comparison is shown. * *q* < 0.05, ** *q* < 0.01.

Ninety-five percent bootstrap confidence intervals (5,000 resamples of the 12 LOSO subjects) for balanced accuracy and macro-averaged F1 score are reported for every decoder × task cell in Supplementary Table S2. Headline values are as follows: GNN weight: 0.643 [0.602, 0.679]; HGBM weight: 0.617 [0.574, 0.657]; CNN surface: 0.565 [0.533, 0.594]; and LogReg surface: 0.562 [0.532, 0.594]. The substantial overlap between the top-performing pair’s confidence intervals is consistent with the FDR-corrected lack of statistical significance for the GNN-*versus*-HGBM (weight) and CNN-*versus*-LogReg (surface) contrasts reported in Table 3 and supports the “match rather than exceed” framing.

### 3.2 Per-subject variability

**Figure 3:**
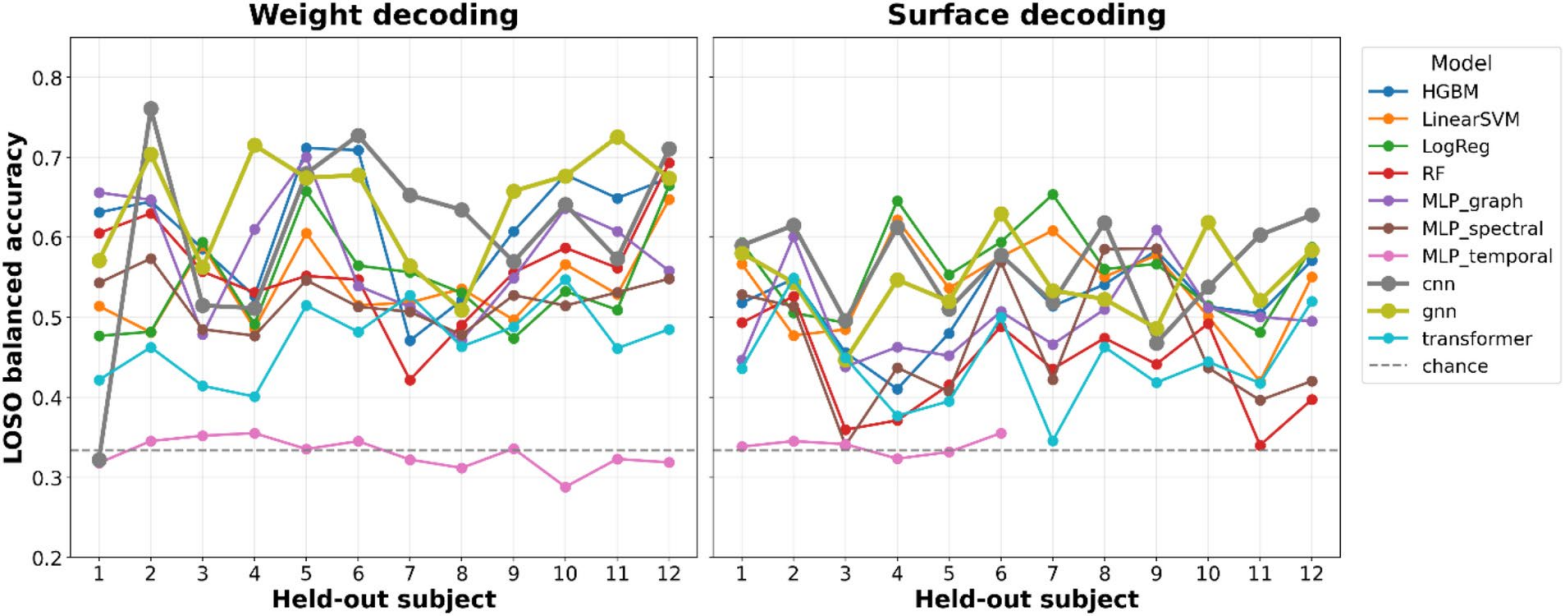
Per-subject LOSO balanced accuracy for each decoder. Each marker represents one of the 12 held-out subjects. The dashed line indicates the chance level (0.333) for the three-class problem. The GNN and CNN are highlighted with thicker lines. The four-layer Transformer trained from scratch (cyan) is consistently below the classical envelope, illustrating its parameter–data mismatch at this clinical sample size.

Figure 3 shows the per-subject LOSO accuracies. For weight decoding, the GNN exceeded chance in every held-out participant, with the highest accuracy for P11 (0.725) and the lowest for P8 (0.509). The CNN showed greater between-subject variability than the GNN or HGBM (range, 0.32 (P1) to 0.76 (P2)), suggesting greater sensitivity to subject-specific differences in electrode placement or normalization. The Transformer remained consistently below the classical envelope on both tasks. The temporal MLP remained close to chance across subjects. Multi-seed robustness is listed as a subsequent analysis in Section 4.6.

### 3.3 Confusion structure

**Figure 4:**
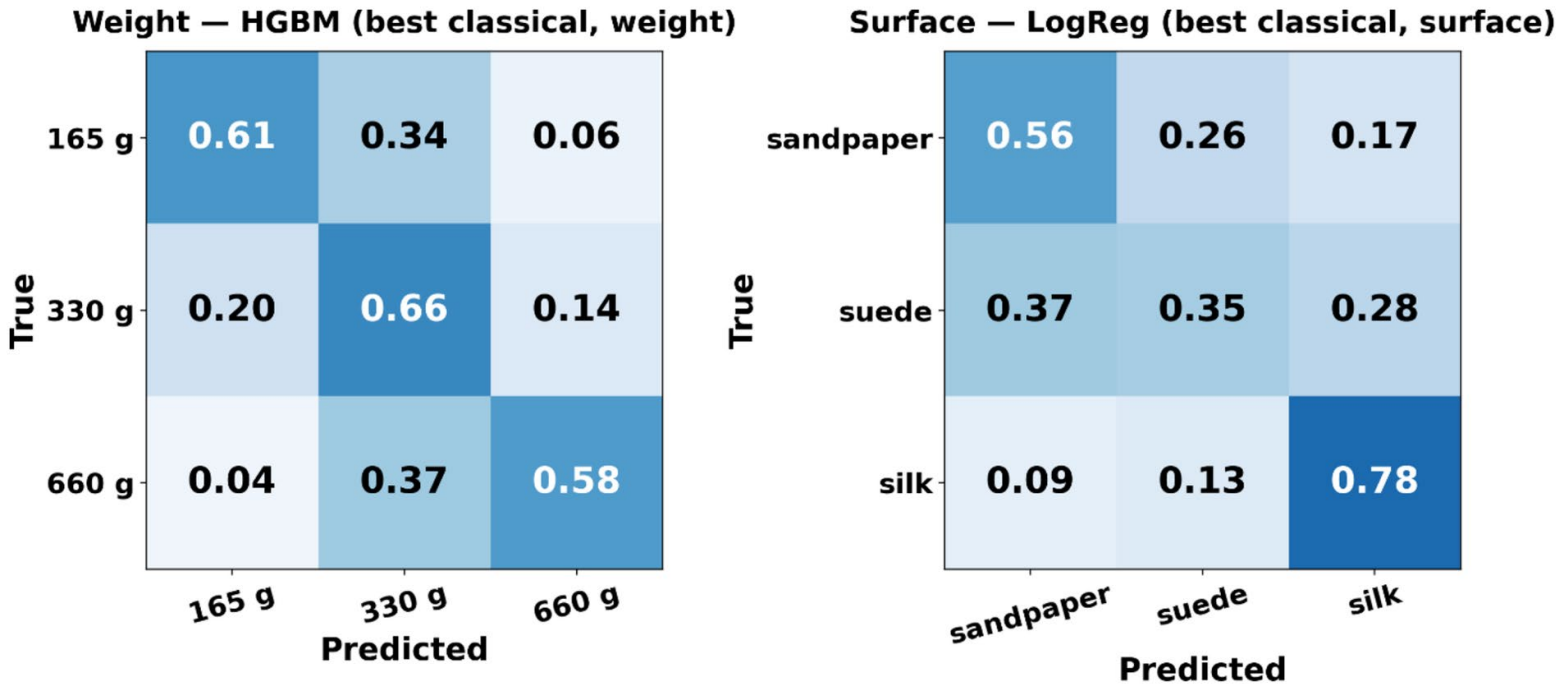
LOSO confusion matrices for the best classical decoder for each task, normalized by the true-class row. Left: weight decoding by HGBM (best classical decoder, 0.617). Right: surface decoding by logistic regression (best classical, 0.562). Class-balanced loss prevents overprediction of the dominant class on the heavily imbalanced surface task.

Figure 4 shows the normalized confusion matrices for the best classical decoder for each task, with predictions concatenated across the 12 LOSO folds. Weight decoding by HGBM was asymmetric: the 660-g class showed the highest sensitivity, and errors were concentrated between adjacent weight levels. Surface decoding by logistic regression was weakest for suede, the least frequent surface class, and the confusions were distributed across silk and sandpaper. The GNN and CNN produced qualitatively similar confusion patterns.

Eight LOSO confusion matrices (HGBM, logistic regression, EEGNet CNN, and GNN, each for weight and surface; predictions concatenated across all 12 folds and row-normalized) are provided in Supplementary Figure S2. Diagonal entries provide the per-class recall directly. In terms of weight, the EEGNet CNN had the highest recall for the 165-g and 660-g classes (0.71 and 0.75), and the GNN showed the most balanced row structure. For surface, HGBM had the highest recall for the silk class (0.87), but the lowest recall for the suede class (0.10), whereas the EEGNet-style CNN showed the most balanced surface profile (0.69/0.26/0.73 for sandpaper/suede/silk).

### 3.4 Fused-model feature importance suggests sensorimotor EEG involvement; modality ablation (Section 3.5.3) shows dominant EMG dependence

**Figure 5:**
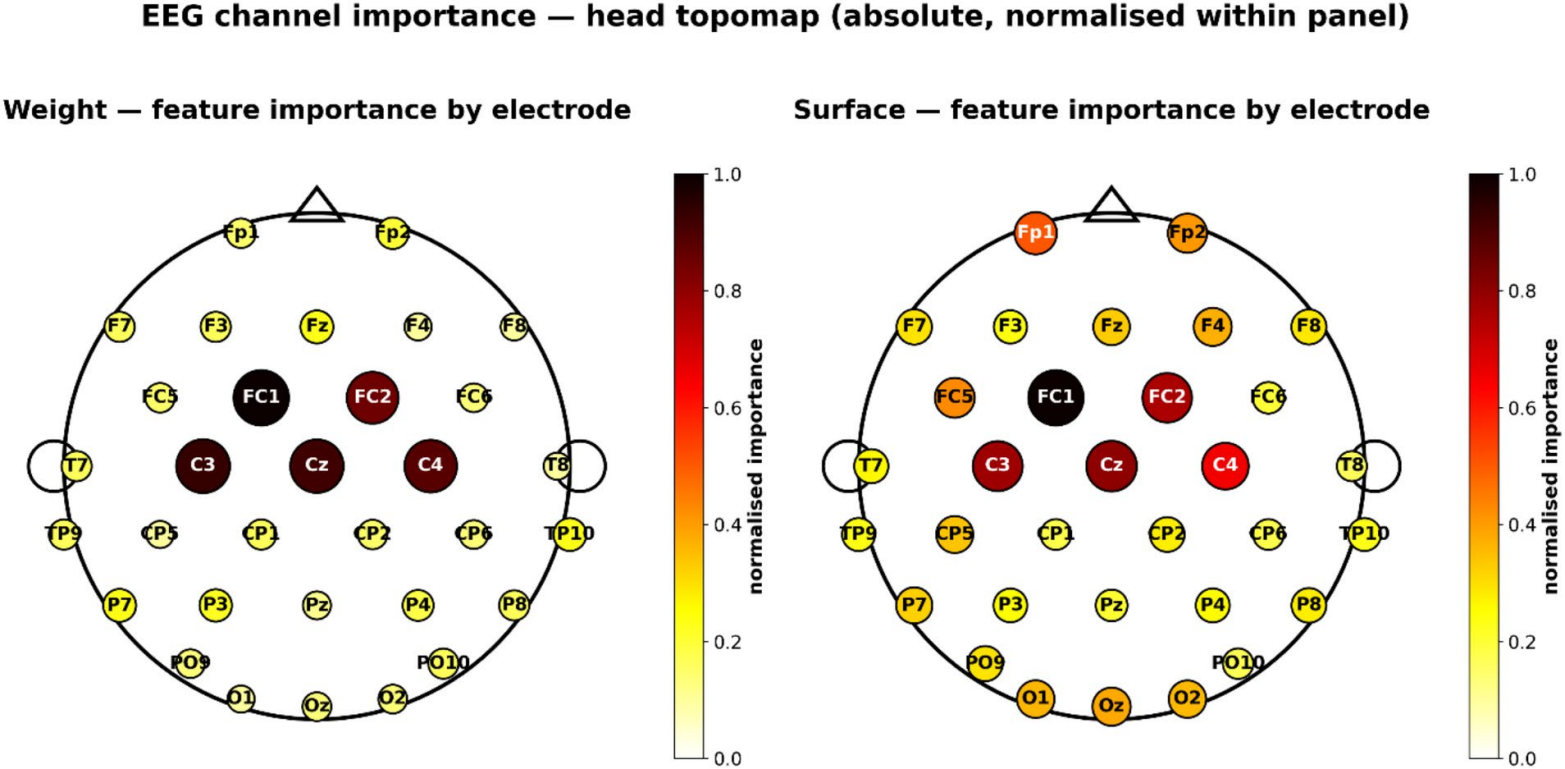
Per-electrode summed feature importance for weight and surface decoding in the fused classical feature space. Marker size and color indicate the summed importance across all band-power and coherence features involving each electrode. Surface-related feature weights were highest over the bilateral sensorimotor electrodes (FC1, FC2, C3, Cz, and C4), but the modality-ablation analysis in Section 3.5.3 showed that these EEG features did not support independent EEG-only decoding. Weight decoding showed weaker EEG involvement and was dominated by EMG features (not shown on the scalp map).

Feature importance in the fused classical models initially suggested different contributions of EMG and EEG (Table 6). For weight decoding, the six highest-ranked features were all EMG band-power features: AD high gamma, brachioradialis low gamma, FDI high gamma, brachioradialis high gamma, flexor digitorum high gamma, and CED high gamma. No EEG features appeared among the top 10 weight features. For surface decoding, the highest-ranked individual features included both EEG and EMG features: Fp1 high gamma, AD high gamma, CED high gamma, FDI high gamma, FC5 high gamma, and O2 high gamma. When importance was aggregated across features involving each EEG channel (Figure 5), the largest surface-related values were observed at FC1, Cz, C3, FC2, and C4. These fused-model maps indicate that sensorimotor EEG features covary with the classification problem, but the modality-ablation analysis in Section 3.5.3 shows that they do not provide strong independent EEG-only decoding.

**Table 6:** Top six features per task by model-native importance.

Rank	Weight feature	Weight importance	Surface feature
1	EMG anterior deltoid—high gamma power	0.061	EEG Fp1—high gamma power
2	EMG brachioradialis—low γ power	0.056	EMG anterior deltoid—high gamma power
3	EMG FDI—high gamma power	0.044	EMG common extensor digitorum—high gamma power
4	EMG brachioradialis—high gamma power	0.036	EMG FDI—high gamma power
5	EMG flexor digitorum—high gamma power	0.013	EEG FC5—high gamma power
6	EMG common extensor digitorum—high gamma power	0.008	EEG O2—high gamma power

### 3.5 Robustness analyses

#### 3.5.1 EMG bandwidth harmonization: the GNN weight advantage depends on bandwidth

The deep models received 20–250 Hz EMG after decimation to 500 Hz, whereas the primary classical spectral baseline used EMG band-power features only up to 95 Hz. Because the 95–250 Hz band can contain motor-unit action-potential shape information related to force production (Pereira et al., 2010), this difference could favor the deep models. We therefore low-pass filtered the EMG channels to 95 Hz and re-ran LOSO cross-validation for the CNN and GNN. The GNN decreased from 0.643 ± 0.071 to 0.568 ± 0.085 on weight decoding, a decrease of 0.074 that was significant after FDR correction (W = 7, d_z_ = 0.96, raw *p* = 0.0093, *q* = 0.033). The CNN was essentially unchanged (0.608 ± 0.121 to 0.622 ± 0.067, *p* = 1.0). After low-pass filtering, the comparison between GNN_harm95 and HGBM reversed direction (Delta = −0.049, d_z_ = −0.64, raw *p* = 0.064), placing HGBM nominally above the GNN under bandwidth-matched inputs. Surface comparisons were unaffected by harmonization.

These findings indicate that the original GNN weight advantage depended on high-frequency EMG content that the primary classical feature set lacked. This result is consistent with the feature-importance analysis, which identified high-gamma EMG amplitude as the main source of weight information. In contrast, the CNN was less sensitive to bandwidth harmonization, suggesting a less concentrated dependence on the high-frequency EMG range.

*Symmetric bandwidth control (exploratory)*. We also tested the opposite harmonization direction by extending the classical HGBM feature set to the same 245-Hz Nyquist bandwidth available to the deep models. Two additional EMG band-power features (95–150 Hz and 150–245 Hz) were added, increasing the classical feature set from 248 to 258 while leaving all hyperparameters unchanged. With this extended feature set, HGBM weight decoding increased from 0.617 ± 0.074 to 0.658 ± 0.081 (Delta = +0.040, d_z_ = 0.54, raw *p* = 0.052). HGBM surface decoding was unchanged (0.518 *vs*. 0.519, *p* = 0.97). The extended-bandwidth HGBM was statistically tied with the original-bandwidth GNN on weight decoding (0.658 *vs*. 0.643; Delta = −0.015, d_z_ = −0.17, *p* = 0.38). Thus, under either harmonization direction, the nominal GNN advantage on weight decoding is better explained by input bandwidth than by a robust architectural advantage.

#### 3.5.2 Early hold *versus* late hold: GNN weight signal concentrates near lift-off

We split the 1-s sustained-hold window into the first 500 ms (early hold) and the last 500 ms (late hold), tiled each segment back to 500 samples to preserve the fixed input length, and re-ran LOSO with the CNN and GNN. This tiling procedure may introduce a low-frequency periodicity artifact, which is acknowledged in Section 4.4. Surface decoding did not differ between early and late hold for either model (CNN: 0.558 *vs*. 0.573, *p* = 0.57; GNN: 0.556 *vs*. 0.542, *p* = 0.30). Weight decoding was also stable for the CNN (0.618 *vs*. 0.611, *p* = 0.73). In contrast, GNN weight decoding was higher during early hold than during late hold (0.639 ± 0.063 *vs*. 0.540 ± 0.080; Delta = 0.098, d_z_ = 1.14, raw *p* = 0.0015, *q* = 0.0066). This finding suggests that the GNN weight signal is strongest soon after lift-off, when grip-force regulation is more dynamic. All eight robustness comparisons from Sections 3.5.1 and 3.5.2 (four bandwidth and four phase contrasts), together with the two exploratory symmetric-extension HGBM contrasts, are summarized in Table 4.

#### 3.5.3 Modality ablation: both tasks decode from EMG; EEG dilutes fusion at clinical sample sizes

To quantify the contribution of each modality, we re-ran HGBM, CNN, and GNN under three input configurations: EEG-only (32 channels), EMG-only (5 channels), and fused EEG-EMG (37 channels). The analysis produced three findings (Table 5).

First, EEG-only decoding was at or near chance for both tasks across all three decoders (weight: 0.34–0.37; surface: 0.36–0.38). All EEG-only *versus* EMG-only comparisons were significant after BH-FDR correction within the modality-ablation family (*q* < 0.0015). Thus, although the fused-model feature-importance map assigned surface-related weights to sensorimotor EEG channels (FC1, Cz, C3, FC2, and C4), these channels did not support independent EEG-only decoding. The fused-model map should therefore be interpreted as reflecting EEG-EMG covariation within the full feature space, rather than independent cortical decoding capacity.

Second, EMG-only decoding produced the highest absolute accuracy in the study. HGBM EMG-only reached 0.712 ± 0.110 for weight and 0.584 ± 0.063 for surface. Adding EEG to EMG reduced performance for HGBM weight decoding (0.712 *vs*. 0.685, *q* = 0.007) and CNN surface decoding (0.606 *vs*. 0.564, *q* = 0.012). Thus, in this dataset and at this sample size, adding EEG channels increased dimensionality without adding sufficient independent task-relevant information to improve these dominant-modality classifiers.

Third, the GNN was robust to EEG-induced dilution. GNN EMG-only and fused performance were statistically equivalent for weight (*q* = 0.60) and surface (*q* = 0.62), and fused performance was nominally higher than EMG-only performance for surface. This pattern is consistent with attention-based soft channel selection: the model can downweight low-information EEG channels rather than forcing all channels through a fixed spatial filter. This contrasts with HGBM, which evaluates all features at each tree split, and with the CNN, which combines all channels via depthwise spatial convolution (Chopra et al., 2026).

These results preserve the architecture-data matching principle but refine its interpretation. In WAY-EEG-GAL, the relevant matching is not a simple peripheral-*versus*-central distinction between weight and surface. Instead, both tasks are predominantly peripheral at the sensor level, and the key architectural property is robustness to low-information channel dilution.

*Note on the modality-ablation feature set*: HGBM in Table 5 used a per-channel summary feature set (mean, standard deviation, interquartile range, and log-band-power in the same four bands used in the primary spectral set), rather than the coherence and log-power feature set used in Table 2. This choice was necessary because EEG-EMG coherence is undefined when one modality is removed. The simpler feature set yielded higher fused weight accuracy in HGBM (0.685) than the primary spectral representation (0.617), suggesting that time-domain summaries added useful information. This observation reinforces the main conclusion: the apparent advantage of deep models depends heavily on the feature set used as a comparator. The CNN and GNN in Table 5 used the same raw 500-sample input as in Table 2, with only the channel set changed.

#### 3.5.4 Partial-crossing conditioning analysis

To test whether the unconditioned decoding accuracies in Section 3.1 were artifacts of the partially crossed weight × surface design, we re-ran CNN and GNN decoding within levels of the conditioning factor. Two subsets preserved the full three-class problem and were most informative: weight decoding within silk (the only surface containing all three weights, *n* = 2,647 trials) and surface decoding within 330 g (the only weight containing all three surfaces, *n* = 1,836 trials). Two additional subsets reduced the problem to two classes but still tested within-condition signal: weight decoding within sandpaper (165 *vs*. 330 g, *n* = 608 trials) and surface decoding within 165 g (sandpaper *vs*. silk, *n* = 1,008 trials). Per-subject means, paired differences relative to the unconditioned (Section 3.1) values, raw *p*-values, and Cohen’s d_z_ for all four conditioning cells (within silk, within 330 g, within sandpaper, and within 165 g) are reported in Table 7.

**Table 7:** Partial-crossing conditioning analysis: within-condition LOSO balanced accuracy for the CNN and GNN, with paired Wilcoxon comparisons to the unconditioned (Section 3.1) values.

Task	Model	Conditioning	Classes	Conditioned accuracy	Unconditioned accuracy	Δ/p
Weight	CNN	Within silk	3	0.636 ± 0.089	0.608	+0.028/0.15
Weight	GNN	Within silk	3	0.634 ± 0.098	0.643	−0.009/0.57
Surface	CNN	Within 330 g	3	0.623 ± 0.095	0.565	+0.059/0.064
Surface	GNN	Within 330 g	3	0.607 ± 0.077	0.544	+0.063/0.009*
Weight	CNN	Within sandpaper	2	0.736 ± 0.105	(n/a)	Chance 0.5
Weight	GNN	Within sandpaper	2	0.766 ± 0.143	(n/a)	Chance 0.5
Surface	CNN	Within 165 g	2	0.884 ± 0.059	(n/a)	Chance 0.5
Surface	GNN	Within 165 g	2	0.866 ± 0.100	(n/a)	Chance 0.5

Within silk and within 330 g preserve the full three-class outcome (chance = 0.333), whereas within sandpaper and within 165 g reduce the problem to two classes (chance = 0.5). The within-330 g GNN surface-decoding result is the only conditioning analysis with a raw *p*-value below 0.01. *raw *p* < 0.01 (uncorrected); conditioning analyses were not included in the FDR-corrected families.

For three-class weight decoding within silk, the CNN reached 0.636 ± 0.089 (*vs*. 0.608 unconditioned; d_z_ = 0.35, raw *p* = 0.15), and the GNN reached 0.634 ± 0.098 (*vs*. 0.643 unconditioned; d_z_ = −0.13, *p* = 0.57). For three-class surface decoding within 330 g, the CNN reached 0.623 ± 0.095 (*vs*. 0.565 unconditioned; d_z_ = 0.65, raw *p* = 0.064), and the GNN reached 0.607 ± 0.077 (*vs*. 0.544 unconditioned; d_z_ = 0.85, raw *p* = 0.009). Thus, conditioning preserved or increased the decoded signal in the full three-class subsets. The two binary subsets also showed clear within-condition information: weight within sandpaper was decoded at 0.736 (CNN) and 0.766 (GNN), and surface within 165 g was decoded at 0.884 (CNN) and 0.866 (GNN), both above the chance level of 0.5.

These results substantially reduce concerns about the partially crossed design. The primary decoding accuracies are not explained solely by the unbalanced cross-feature design; conditioning on one level of the other factor preserved or modestly improved the decoded signal. The architecture-data matching interpretation is therefore robust to the partially crossing structure of WAY-EEG-GAL.

#### 3.5.5 Deep-model interpretability sanity check

To address whether the compact deep models learned structured signal representations, we added a descriptive model-native interpretability analysis (Supplementary Figure S1). The EEGNet first-block temporal filters showed structured frequency responses, with prominent energy in the beta and high gamma bands and secondary energy at lower frequencies. The EEGNet depthwise spatial weights emphasized bilateral sensorimotor EEG channels and relatively high EMG-channel weights. For the GNN, attention rollout averaged across heads, held-out trials, and LOSO folds showed that attention received by EMG nodes was higher than the mean attention received by EEG channels, consistent with the modality-ablation result that task-relevant information was concentrated in EMG. These analyses do not replace the modality-ablation analysis as causal evidence of modality relevance, but they provide a model-native sanity check that the compact deep models learned structured temporal filters and channel-weighting patterns.

## 4 Discussion

### 4.1 Compact deep models match—but do not significantly exceed—classical decoders at clinical sample sizes

The main result is that compact deep models matched, but did not significantly outperform, well-engineered classical decoders. Without bandwidth controls, neither deep-*versus*-best-classical comparison reached statistical significance after Benjamini–Hochberg FDR correction. After EMG bandwidth harmonization, the GNN weight advantage reversed direction, with HGBM nominally exceeding the low-pass-filtered GNN. The CNN remained stable across bandwidth conditions. Thus, the deep models did not fail at clinical sample sizes, but they also did not provide a transformative gain over classical baselines. This agrees with the broader BCI literature, in which compact deep models tend to show their clearest advantage when training datasets are sufficiently large for classical decoders to plateau.

Three findings nevertheless support an architecture-relevant interpretation. First, the GNN outperformed its no-attention MLP analog on weight decoding (*q* = 0.030), indicating that attention-weighted message passing contributed measurable information. Second, the four-layer Transformer trained from scratch (approximately 600 k parameters) underperformed on both tasks (*q* = 0.003), consistent with a mismatch between the model’s capacity and the training set size. Third, the temporal MLP remained near chance, showing that raw multichannel temporal data require either feature engineering or a suitable inductive bias.

### 4.2 Both tasks are peripherally encoded; EEG dilutes fusion at *n* = 12

The modality-ablation analysis refined the interpretation of the feature-importance analysis. Across HGBM, CNN, and GNN, EEG-only decoding was at or near chance for both tasks, whereas EMG-only decoding produced the highest absolute performance. Thus, both weight and surface decoding were predominantly peripheral in this dataset and at this sample size. The sensorimotor EEG features highlighted in the fused classical surface decoder should be interpreted as EEG features that covaried with the EMG-driven classification problem, rather than as evidence for independent EEG-only surface decoding. Cortical signatures of grasp-surface friction remain physiologically plausible, but they were not recoverable here with sensor-level EEG, a 1-s sustained-hold window, and 12 participants.

Fusion dilution provides the second important result. Adding 32 EEG channels to the five EMG channels reduced performance for HGBM weight decoding and CNN surface decoding. In contrast, the GNN tolerated the additional EEG channels without loss. A plausible mechanism is soft channel selection: the GNN can learn attention weights that reduce the impact of low-information channels, whereas the CNN and HGBM are more directly affected by the enlarged input space.

Therefore, architecture-data matching should be understood here as a match between model inductive bias and the dominant modality. For WAY-EEG-GAL, both decoded variables are mainly carried by EMG at the sensor level. Under these conditions, one can either restrict input to the relevant modality or use an architecture, such as graph attention, that can suppress uninformative channels. Denser EEG, source-localized signals, longer windows that include pre-contact tactile transients, or task-specific fine-tuning may reveal EEG-driven friction signatures that were not detected in the present analysis.

### 4.3 The transformer underperforms—but lighter convolution–attention hybrids may not

A complementary and FDR-robust finding is that our four-layer Transformer trained from scratch (approximately 600 k parameters) underperformed both classical and graph-based decoders on both tasks (*q* = 0.003). With 11 training subjects × approximately 294 trials per subject (approximately 3,234 training trials per LOSO fold), the Transformer’s parameter-to-training-trial ratio is approximately 185:1, well above the empirical threshold at which large attention models reliably overfit in this data regime. The approximately 50 k-parameter GNN (ratio ≈ 15:1) and the approximately 4 k-parameter EEGNet (ratio ≈ 1:1) operate in regimes where their inductive biases dominate over their parameter budgets.

This finding is specific to the particular Transformer configuration we evaluated and should not be interpreted as evidence against attention itself. A growing class of compact convolution–attention hybrids—EEG-Conformer (Song et al., 2023), DBConformer (Wang et al., 2026), EEG-MFTNet (Andrikopoulos and Mehrkanoon, 2026), and channel-token temporal-attention GNNs such as EEG-tGAT (Chopra et al., 2026)—places a single attention block after a convolutional stem, controlling the parameter budget to approximately 30 k–100 k parameters while preserving attention’s relational structure. These models report gains over EEGNet on motor-imagery datasets at comparable sample sizes and would be the appropriate comparators for any conclusion about “attention as such” at the clinical scale. The release of revision_ablations.py (Section 4.6) includes a lightweight Conformer-style hybrid that reviewers may use as a tighter attention baseline; in the present submission, we report the four-layer Transformer trained from scratch as a single, transparent data point on the parameter–data trade-off.

Overfitting at this data scale is the most likely explanation for the underperformance of the four-layer Transformer trained from scratch. The class-balanced loss, EEGNet stem batch normalization, dropout in every architecture, AdamW weight decay, and the fixed 30-epoch schedule (to avoid excessive training) were deliberate choices intended to mitigate this risk. The per-subject SDs in Figure 3 are informative: the Transformer’s per-subject SD is the smallest among the deep models, consistent with uniform overfitting to the training distribution rather than idiosyncratic fitting of particular subjects. We have therefore narrowed the conclusion to apply specifically to the four-layer Transformer trained from scratch at this data scale, not to attention-based deep models in general; lighter convolution–attention hybrids such as EEG-Conformer (Song et al., 2023) are the appropriate comparator for attention at the clinical scale and are identified as a subsequent analysis in Section 4.6.

### 4.4 Limitations

Seven limitations should be considered:

First, the sample size of 12 participants is small for deep-learning applications. Absolute accuracy values should therefore be interpreted as within-dataset comparisons rather than as transferable benchmarks. The Transformer result is also specific to this data scale and training regime.

Second, deep models were evaluated with a single random seed (seed = 0). Although between-subject variability is usually larger than seed-to-seed variability in small EEG datasets, a five-seed re-run would provide more stable confidence intervals. This analysis is prioritized in the subsequent released analysis.

Third, hyperparameters were taken from canonical implementations rather than optimized with nested cross-validation. Nested LOSO at *n* = 12 would leave very small inner training sets, and fixed defaults are common in this literature (Roy et al., 2019). Nevertheless, modest sweeps over HGBM depth, dropout, and learning rate could slightly change the ranking. The released code exposes these hyperparameters to support such tests.

Fourth, the WAY-EEG-GAL design is partially crossed (suede only at 330 g; 660 g only with silk; Supplementary Table S1). The conditioning analysis in Section 3.5.4 substantially reduces this concern for the within-task accuracy claims because within-silk weight decoding was equivalent to unconditioned decoding and within-330 g surface decoding was higher for the GNN. However, the modality-ablation analysis should still be interpreted as identifying the dominant signal source rather than proving a fully causal dissociation between object properties.

Fifth, ICA artifact rejection was not applied. This choice preserved identical preprocessing across decoders, but residual artifacts could affect raw-signal deep models differently from classical band-power features. An ICA-*versus*-no-ICA analysis is therefore a useful subsequent analysis.

Sixth, several relevant baselines were not included, including FBCSP (Ang et al., 2012), Riemannian-geometry SPD methods, and compact convolution–attention hybrids. This study should therefore be viewed as a controlled first benchmark rather than a definitive comparison of all available approaches.

Seventh, the 1-s sustained-hold window and center-crop alignment were fixed *a priori*. Other windows (0.5 s or 2.0 s) and anchors (lift-onset aligned or hold centered) may yield different results. In addition, the early-*versus*-late analysis tiled 250-sample segments to preserve input length, which may introduce a small low-frequency periodicity artifact. Finally, feature importance was computed on a model fitted to all 12 subjects rather than averaged across LOSO folds; cross-validated feature importance would reduce this explanatory limitation.

WAY-EEG-GAL comprises healthy adults performing a controlled, cued grasp-and-lift task. The present results address architectural comparison at a small-sample neural-decoding scale; they do not constitute direct evidence for clinical populations, neurorehabilitation settings, or patient-specific decoding pipelines. Throughout this manuscript, “clinical-scale” refers specifically to the data regime (small participant count and high between-subject variability), not to clinical populations.

### 4.5 Implications for clinical-scale neural-decoding studies

Three practical implications follow. First, in clinical-scale neural-engineering datasets, compact deep architectures can match well-tuned classical decoders, but large performance claims should be supported by paired statistics and appropriate correction for multiple comparisons. Second, model capacity must be matched to the sample size; large attention models trained from scratch may underperform when the sample size is small. Third, modality relevance is often more important than channel count. When the informative signal is concentrated in a small subset of channels, performance may improve by selecting the relevant modality or by using architectures, such as graph attention, that can downweight low-information channels.

The present comparison also leaves open an important question at the representation level. We contrasted hand-crafted spectral features with raw temporal tensors, but wavelet and learnable time–frequency representations provide an intermediate inductive bias that may be particularly useful for non-stationary EEG and EMG. Recent RDWT-based preprocessing and learnable wavelet front-end models have shown that time–frequency structure can improve robustness and interpretability in motor-imagery EEG decoding (Siino et al., 2025; Siino et al., 2026). Such approaches could be especially relevant for EEG-EMG decoding because they may preserve transient sensorimotor events while still constraining the model toward physiologically meaningful oscillatory structure. Future studies should therefore compare raw temporal inputs, fixed spectral features, wavelet/scalogram representations, and learnable wavelet front-ends under the same LOSO and modality-ablation framework used here.

**Figure 6:**
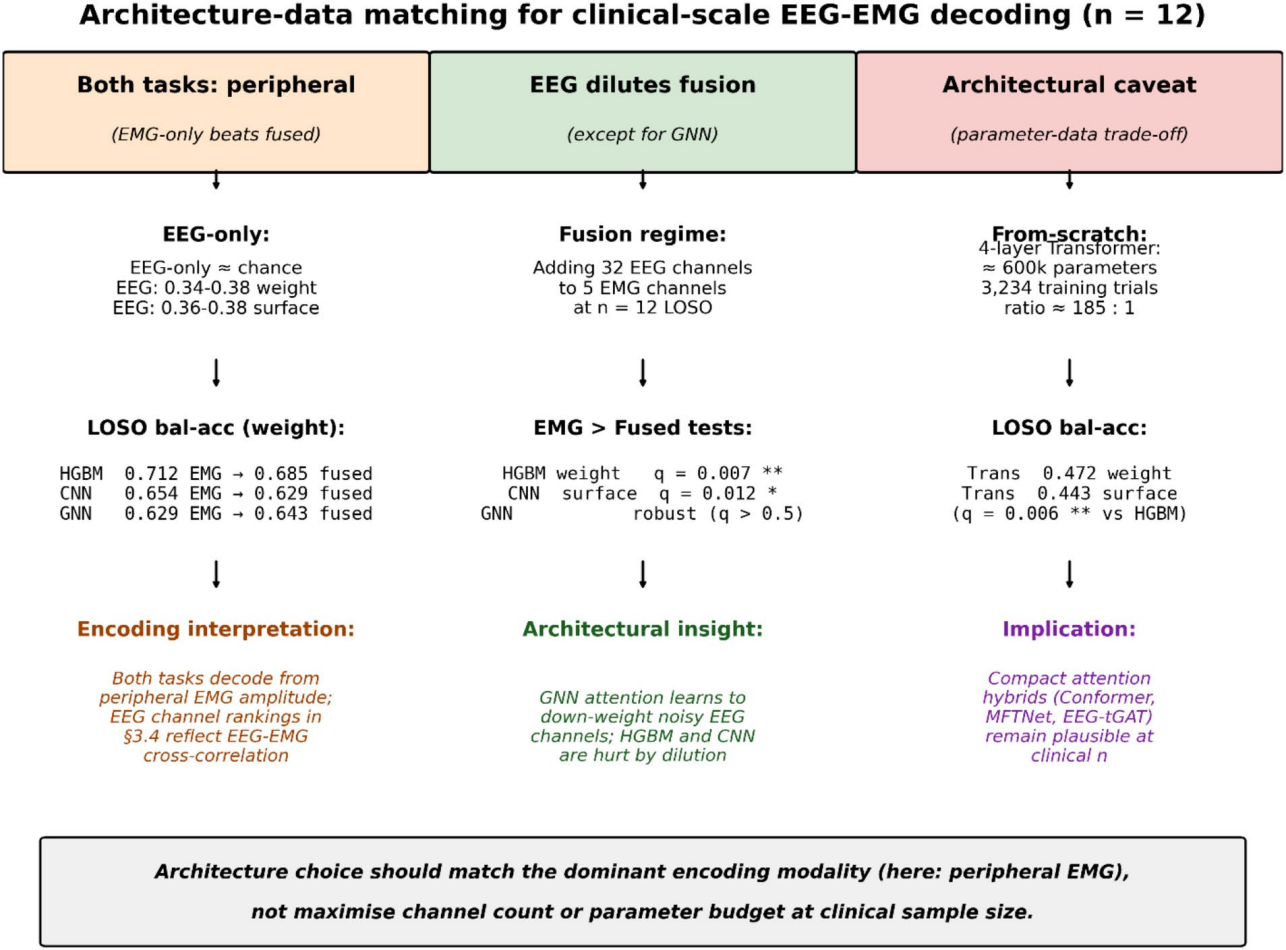
Schematic summary of the architecture-data matching principle. Each column links a decoded variable or architectural caveat to its dominant signal source, best-performing decoder, and corresponding robustness analysis. The central message is that architecture should be chosen to match the informative modality and data scale rather than selected solely based on performance on large external benchmarks.

Figure 6 summarizes the three pillars of the architecture-data matching principle: the dominance of the peripheral modality for both decoded variables, the differential vulnerability of HGBM and the CNN to EEG-induced fusion dilution *versus* the robustness of the GNN, and the parameter–data trade-off underlying the underperformance of the four-layer Transformer trained from scratch at this clinical sample size.

### 4.6 Identified follow-up experiments

Four robustness analyses were completed in this submission: bidirectional bandwidth harmonization (Section 3.5.1, including the exploratory extension of classical EMG features to 245 Hz), early hold *versus* late hold decoding (Section 3.5.2), EEG-only/EMG-only/fused-modality ablation (Section 3.5.3), and partial-crossing conditioning (Section 3.5.4). Six additional analyses are prioritized for future study: (i) five-seed robustness for the CNN, GNN, and Transformer; (ii) a compact convolution–attention hybrid as a lower-parameter attention comparator; (iii) expanded model-native interpretability for the deep decoders, including fold-wise stability of temporal filters, attention weights, and saliency maps; (iv) ICA-*versus*-no-ICA preprocessing; (v) sparse topology-informed GNN adjacency; and (vi) explicit channel-selection mechanisms for HGBM and CNN, such as per-channel gates or group-regularized first layers.

## 5 Conclusion

On the public WAY-EEG-GAL grasp-and-lift dataset, compact deep models matched, but did not significantly outperform, classical spectral decoders under LOSO evaluation. The GNN achieved the highest nominal accuracy for weight decoding, and the EEGNet-style CNN achieved the highest nominal accuracy for surface decoding; neither advantage remained significant after Benjamini–Hochberg FDR correction relative to the best classical baseline. Bidirectional bandwidth control showed that the GNN weight advantage was bandwidth dependent rather than clearly architectural: low-pass filtering of deep-model EMG inputs reduced GNN accuracy from 0.643 to 0.568 (*q* = 0.033), whereas extending classical EMG features to 245 Hz increased HGBM accuracy from 0.617 to 0.658 (raw *p* = 0.052), making it statistically tied with the GNN (*p* = 0.38). Additional robust findings were that the four-layer Transformer trained from scratch underperformed, the raw-temporal MLP remained near chance, and the GNN outperformed its no-attention MLP analog on the weight-decoding task. Feature-importance analysis in fused models initially suggested a peripheral-*versus*-central dissociation, but modality-ablation analysis showed that EEG-only decoding was at chance for both tasks and that both tasks were predominantly EMG-driven at this sample size. Partial-crossing conditioning further showed that the unconditioned decoding accuracies were not explained solely by the unbalanced WAY-EEG-GAL design. Together, the results are consistent with and motivate an architecture-data matching principle for clinical-scale decoding studies. At small sample sizes, the more important design choice may be not architectural complexity itself but whether the model’s inductive bias matches the informative modality and can suppress low-information channels.

## Data Availability

The raw WAY-EEG-GAL dataset analyzed in this study is publicly available from Luciw et al. (2014) and via PhysioNet. All analysis code, derived per-subject feature tensors, model checkpoints, and figure-generation scripts are available on GitHub (https://github.com/osmar235/eeg-emg-architecture-data-matching) and archived on Zenodo (DOI: 10.5281/zenodo.19966634).
