# Supplementary material for: Architecture-data matching for EEG-EMG decoding: compact deep models match classical spectral decoders on the WAY-EEG-GAL grasp-and-lift dataset
Source: Front Neurosci. 2026 Jul 20;20:1874302. doi: 10.3389/fnins.2026.1874302 (PMC13429724; doi:10.3389/fnins.2026.1874302)
Supplement: Supplementary file 1 [file Supplementary_file_1.docx]

Supplementary Material

**Supplementary Table S1. Trial counts per (weight × surface) cell for the WAY-EEG-GAL dataset, summed across all 12 participants.**

The matrix exposes the partial-crossing structure of the dataset: suede (column) appears only with the 330-g weight; 660 g (row) appears only with silk. Within-condition decoding analyses addressing this structure are reported in §3.5.4 and Table 7.

|  | **Sandpaper** | **Suede** | **Silk** |
| --- | --- | --- | --- |
| **165 g** | 168 | 0 | 840 |
| **330 g** | 440 | 273 | 1,123 |
| **660 g** | 0 | 0 | 684 |

Total trials: 3,528. Per-subject trial counts are within ±1 of these totals divided by 12 (sessions are matched across participants).

**Supplementary Table S2. Bootstrap 95% confidence intervals for balanced accuracy and macro-F1 across decoders and tasks.**

Confidence intervals were computed by bootstrap resampling (5,000 resamples) of the 12 leave-one-subject-out per-fold scores, with bal_mean and f1_mean reporting the unweighted mean across folds. The MLP (temporal) result for the surface task rests on six retained folds (the remaining six folds collapsed to a single predicted class on training, producing degenerate predictions; this is a property of the no-inductive-bias control rather than a metric problem). The wide overlap between the top decoders on each task — GNN vs HGBM on weight, and CNN vs LogReg on surface — is consistent with the BH-FDR-corrected non-significance reported in Table 4 and supports the “match rather than exceed” framing in §4.1.

| **Task** | **Decoder** | **Bal-Acc** | **95% CI** | **Macro-F1** | **95% CI** |
| --- | --- | --- | --- | --- | --- |
| surface | LogReg | 0.562 | [0.532, 0.594] | 0.517 | [0.486, 0.547] |
| surface | LinearSVM | 0.539 | [0.506, 0.569] | 0.520 | [0.486, 0.553] |
| surface | RandomForest | 0.436 | [0.403, 0.467] | 0.428 | [0.390, 0.462] |
| surface | HGBM | 0.518 | [0.488, 0.544] | 0.503 | [0.477, 0.528] |
| surface | MLP (spectral) | 0.470 | [0.425, 0.516] | 0.456 | [0.414, 0.495] |
| surface | MLP (channel-pooled) | 0.500 | [0.472, 0.533] | 0.495 | [0.478, 0.514] |
| surface | MLP (temporal) | 0.339 | [0.331, 0.347] | 0.318 | [0.303, 0.334] |
| surface | CNN (EEGNet) | 0.565 | [0.533, 0.594] | 0.516 | [0.481, 0.546] |
| surface | GNN | 0.544 | [0.515, 0.573] | 0.459 | [0.421, 0.489] |
| surface | Transformer | 0.443 | [0.412, 0.476] | 0.394 | [0.357, 0.428] |
| weight | LogReg | 0.544 | [0.510, 0.581] | 0.488 | [0.446, 0.532] |
| weight | LinearSVM | 0.540 | [0.515, 0.569] | 0.496 | [0.466, 0.530] |
| weight | RandomForest | 0.561 | [0.522, 0.596] | 0.562 | [0.513, 0.606] |
| weight | HGBM | 0.617 | [0.574, 0.657] | 0.607 | [0.561, 0.651] |
| weight | MLP (spectral) | 0.520 | [0.504, 0.538] | 0.510 | [0.489, 0.532] |
| weight | MLP (channel-pooled) | 0.580 | [0.540, 0.621] | 0.561 | [0.517, 0.605] |
| weight | MLP (temporal) | 0.329 | [0.318, 0.339] | 0.310 | [0.297, 0.322] |
| weight | CNN (EEGNet) | 0.608 | [0.539, 0.669] | 0.550 | [0.478, 0.609] |
| weight | GNN | 0.643 | [0.602, 0.679] | 0.616 | [0.568, 0.660] |
| weight | Transformer | 0.472 | [0.447, 0.498] | 0.442 | [0.407, 0.474] |

**Supplementary Figure S1. Model-native interpretability sanity check for the compact deep decoders.**


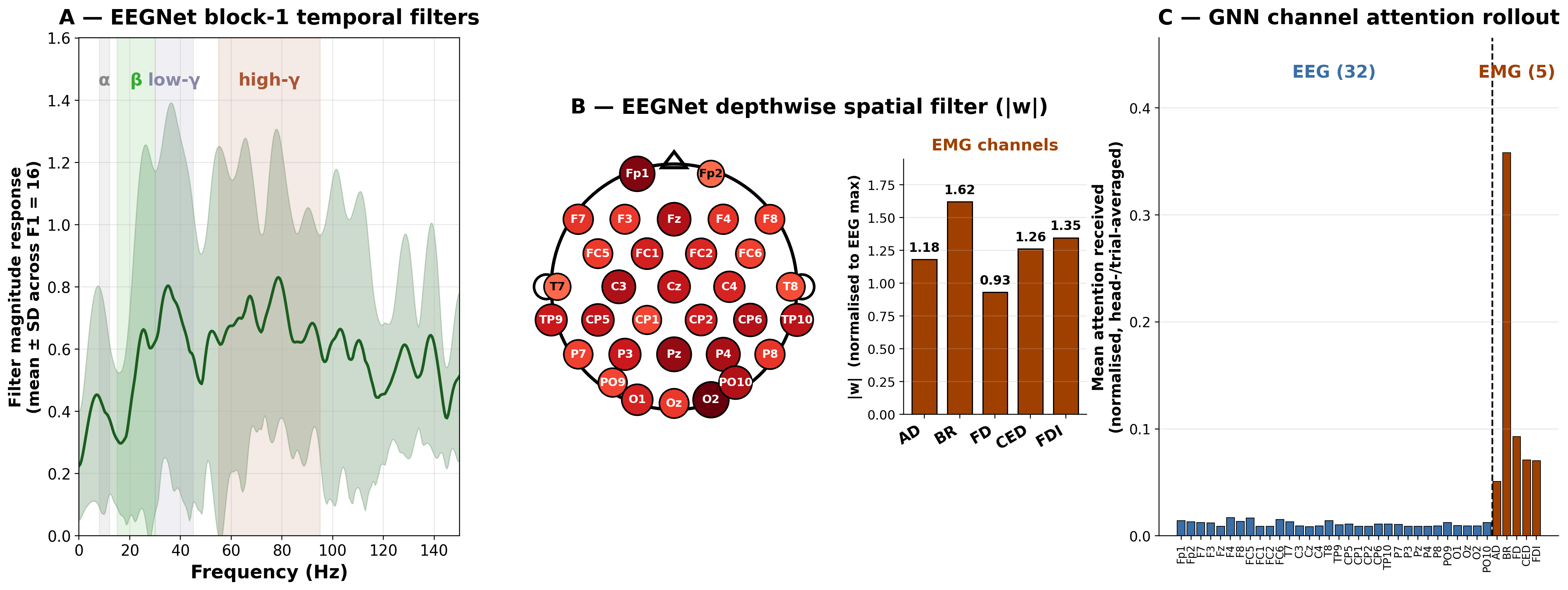


(A) Frequency response of the first temporal convolutional block of the EEGNet-style CNN. Each of the F1 = 16 learned 1 × 64 temporal filters was extracted from the trained models and transformed into a magnitude response using the fast Fourier transform. The solid line shows the mean response across filters, and the shaded area shows ±1 SD. Colored vertical bands indicate the classical spectral feature ranges used in the main analysis: alpha, beta, low-gamma, and high-gamma. The learned filters showed structured responses, with prominent energy across the β and γ ranges (peaks near 30–40 Hz and 60–80 Hz) and secondary energy below 8 Hz, rather than degenerate impulse-like filters.

(B) Absolute depthwise spatial-filter weights from the EEGNet-style CNN. For each channel, the absolute weights were averaged across the F1 · D = 32 depthwise filters. EEG weights were projected onto the 10–20 scalp layout and normalized within the EEG map; EMG-channel weights are shown separately and normalized to the maximum EEG-channel weight. The spatial weights emphasized bilateral sensorimotor EEG channels and showed high relative magnitude for the EMG channels, consistent with the fused-model feature-importance analysis and the modality-ablation result that decoding was dominated by peripheral EMG information.

(C) GNN channel-attention rollout. Channel-level attention was extracted from the two graph-attention layers and averaged across attention heads, held-out trials, and LOSO folds. Bars show the mean attention received by each channel (averaged across heads, layers, held-out trials, and LOSO folds) and then normalized to sum to one across the 37 channels. The vertical dashed line separates EEG channels from EMG channels. Attention was concentrated on the five EMG channels, supporting the interpretation that the GNN learned a soft channel-selection mechanism that down-weighted low-information EEG channels during fused EEG-EMG decoding. These visualizations are descriptive sanity checks and were not included in the formal statistical comparison families.

**Supplementary Figure S2. LOSO confusion matrices for HGBM, logistic regression, the EEGNet CNN, and the GNN.**


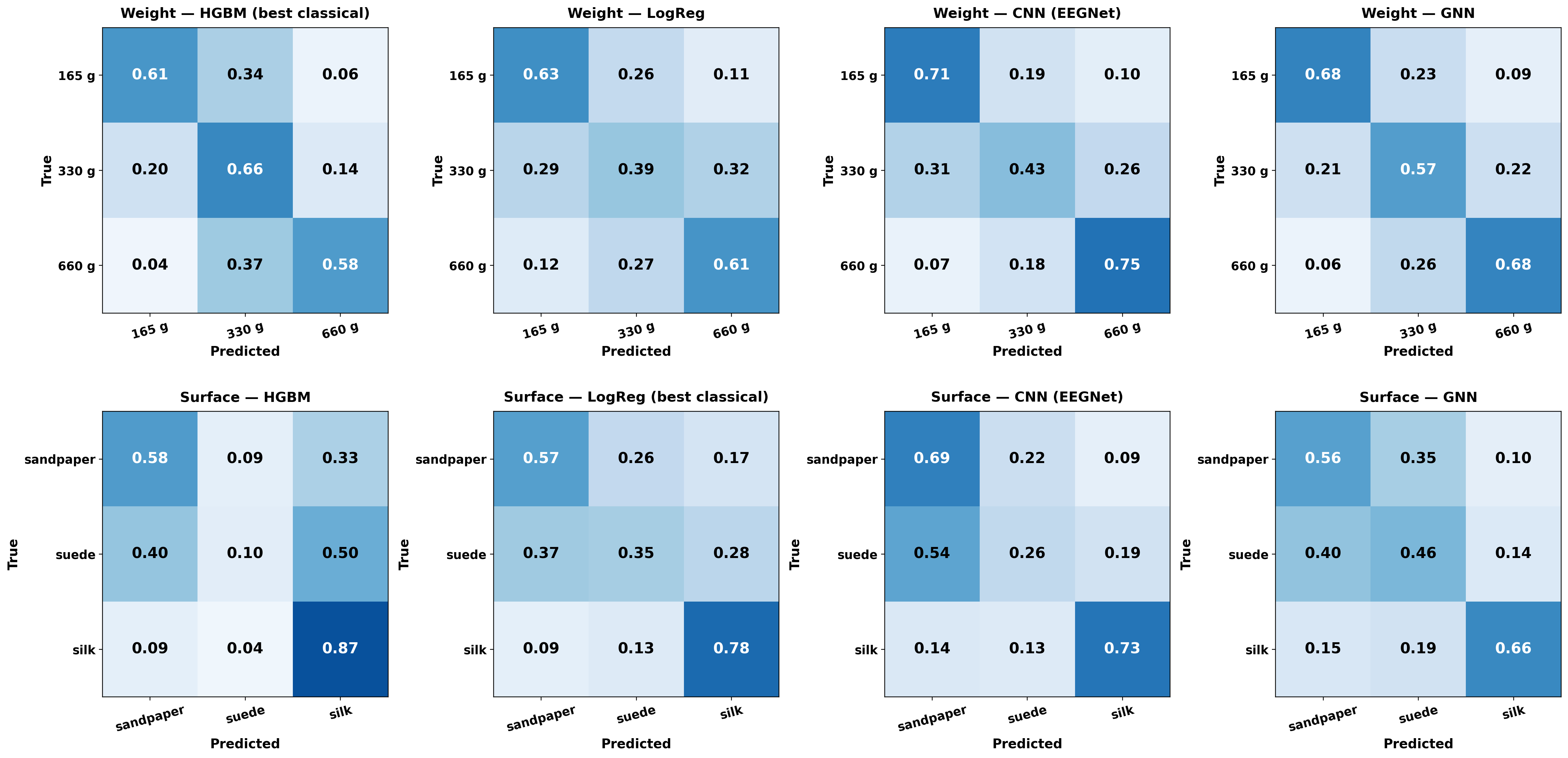


Normalized confusion matrices are shown for weight decoding (top row) and surface decoding (bottom row), with predictions concatenated across the 12 leave-one-subject-out folds. Rows indicate the true class and columns indicate the predicted class; each row is normalized to sum to 1, so diagonal entries correspond to per-class recall/sensitivity. For weight decoding, all four models captured the ordered structure of the task, with errors occurring mainly between adjacent weights; the EEGNet CNN attained the highest 165-g and 660-g recall (0.71 and 0.75), HGBM the highest 330-g recall (0.66), and the GNN the most balanced row structure. For surface decoding, all models showed reduced performance on the minority suede class; HGBM attained the highest silk recall (0.87) but the worst suede recall (0.10), whereas the EEGNet CNN gave the most balanced surface profile (0.69 / 0.26 / 0.73). These class-specific patterns complement the balanced-accuracy and macro-F1 results reported in Table 2 and Supplementary Table S2 and show that the main model comparisons are not driven solely by global accuracy differences.
